# Critical Documents on Cow's Milk Allergy and Anaphylaxis: Two Recent WAO Position Papers

**DOI:** 10.1097/WOX.0b013e31821e9e45

**Published:** 2011-05-15

**Authors:** Lanny J Rosenwasser

**Affiliations:** 1 Editor-in-Chief

## 

The purpose of this editorial is to highlight the recent ratification as WAO Position Papers of 2 important documents published in the *World Allergy Organization Journal *(*WAO Journal*).

The "World Allergy Organization (WAO) Guidelines on the Diagnosis and Rationale against Cow's Milk Allergy (DRACMA) Guidelines," ratified as a WAO Position Paper by the WAO House of Delegates, March 21, 2011, involves a significant pediatric food allergy problem and provides invaluable guidelines for the clinician. This impressive collection of information on this worldwide problem will help in the management of cow's milk allergy, which has profound implications for growth and development and the health of children worldwide. Under the able leadership of Professor Alessandro Fiocchi (Italy), the Chair of the WAO Special Committee on Food Allergy, an international group has put together a scholarly and well-appointed review of all the issues related to cow's milk allergy (CMA), ranging from methodology and epidemiology through a variety of other aspects of the clinical syndrome. DRACMA provides the guidelines for diagnosing CMA, and the proper natural history treatment alternatives ranging from avoidance through identification of milk derived from a variety of other sources, including unusual sources, which can be used for dietary supplementation. DRACMA employed evidence-based methodology utilizing the criteria of Grading of Recommendations Assessment, Development and Evaluation (GRADE) in its analysis of the literature.

"World Allergy Organization Guidelines for the Assessment and Management of Anaphylaxis," the WAO Position Paper ratified on February 18, 2011, is a thorough, extensive work completed by worldwide anaphylaxis and allergy experts of the WAO Special Committee on Anaphylaxis led by Chair, Professor F. Estelle Simons. In addition to clearly defining, in a concise manner, the syndrome, its severity, and the possibility for treatment, the document has also provided a very clear and detailed management plan for acute anaphylaxis in both high- and low-resource areas. This is an important stride forward and the *WAO Journal *as always looks to better care for allergy sufferers at risk for anaphylaxis and for significant morbidity and mortality. This position paper is an excellent resource to help those circumstances.

The Position Paper series within the *WAO Journal *is intended to address the needs of the profession worldwide. Along with the 2 most recent documents, and another on the state of sublingual immunotherapy (SLIT), the series also includes a treatise on defining an allergist, basic qualities of an allergist, training required for allergists, and educational requirements--all to impart allergy education to students, residents, and practitioners. The WAO and the *WAO Journal *will continue to invest in the development and identification of guidelines for treating important, clinically relevant syndromes associated with allergic disease such as cow's milk allergy or anaphylaxis.

The recently ratified position papers and other WAO documents published in *WAO Journal *can be accessed in the following linked list of titles:

• World Allergy Organization (WAO) Diagnosis and Rationale for Action against Cow's Milk Allergy (DRACMA) Guidelines

• World Allergy Organization Guidelines for the Assessment and Management of Anaphylaxis

• Sub-Lingual Immunotherapy: World Allergy Organization Position Paper 2009

• Recommendations for Competency in Allergy Training for Undergraduates Qualifying as Medical Practitioners: A Position Paper of the World Allergy Organization

• Requirements for Physician Competencies in Allergy: Key Clinical Competencies Appropriate for the Care of Patients With Allergic or Immunologic Diseases: A Position Statement of the World Allergy Organization

• What Is an Allergist?: Reconciled Document Incorporating Member Society Comments, September 3, 2007

